# Role of Sex in the Therapeutic Targeting of p53 Circuitry

**DOI:** 10.3389/fonc.2021.698946

**Published:** 2021-07-08

**Authors:** Francesca Mancini, Ludovica Giorgini, Emanuela Teveroni, Alfredo Pontecorvi, Fabiola Moretti

**Affiliations:** ^1^ Research Unit on Human Reproduction, International Scientific Institute Paul VI, Fondazione Policlinico A. Gemelli, IRCCS, Rome, Italy; ^2^ Institute of Biochemistry and Cell Biology, National Research Council of Italy, Monterotondo, Italy; ^3^ Catholic University of the Sacred Heart of Rome, Fondazione Policlinico A. Gemelli, IRCCS, Rome, Italy

**Keywords:** cancer therapy, p53, sex, estrogen, MDM2, MDM4

## Abstract

Sex profoundly affects cancer incidence and susceptibility to therapy, with sex hormones highly contributing to this disparity. Various studies and omics data suggest a relationship between sex and the oncosuppressor p53 circuitry, including its regulators MDM2 and MDM4. Association of this network with genetic variation underlies sex-related altered cancer risk, age of onset, and cancer sensitivity to therapy. Moreover, sex-related factors, mainly estrogenic hormones, can affect the levels and/or function of the p53 network both in hormone-dependent and independent cancer. Despite this evidence, preclinical and clinical studies aimed to evaluate p53 targeted therapy rarely consider sex and related factors. This review summarizes the studies reporting the relationship between sex and the p53 circuitry, including its associated regulators, MDM2 and MDM4, with particular emphasis on estrogenic hormones. Moreover, we reviewed the evaluation of sex/hormone in preclinical studies and clinical trials employing p53-target therapies, and discuss how patients’ sex and hormonal status could impact these therapeutic approaches.

## Introduction

Cancer statistics reveal sex (meant as biological factors) differences in incidence, therapy response, and mortality of many cancers (1). The majority of these tumors, excluding sex-related prostate ovary and breast cancer, present a higher incidence, increased invasive property, and cancer death in males compared to female, even after correction for environmental exposures and risk habits, as smoking and alcohol consumption, more common among men (2). Primarily, genetic factors residing on sex chromosomes contribute to these disparities (3). Indeed, the X chromosome contains various genes involved in oncogenesis (4). Since a percentage of genes is not silenced in inactivated X-chromosome, their higher expression levels in females compared to males may underlie cancer differences (4, 5).

Additionally, an important factor contributing to sexual differentiation is the circulating sex hormones with a strong relevance of estrogen (6, 7). Women have a higher risk of developing lung cancer upon smoking than men. Various studies suggest that the interaction between tobacco carcinogens and endogenous and exogenous sex steroids may be relevant (8). Nonetheless, this disparity persists among adults aged 85 and older, thus beyond the women’s reproductive age (9). The levels of intra-tissue sex hormones determined by local production of the estrogen (intracrinology) are assuming great importance in many hormone-related tumors (10, 11). Finally, epigenetic modification of DNA is different in the two sexes with DNA methylation enhanced in various organs of experimental feminine rodents. Since cancer is linked to epigenetic dysregulation, these differences play an important function, too [reviewed in (5)].

All these factors can variously affect molecular pathways involved in oncogenesis. The p53 pathway is one of the most relevant in tumor development and therapy response, and p53 is a crucial oncosuppressor in both humans and rodents (12, 13). TP53 gene is mutated in about 50% of human cancers, while the protein is inactivated in tumors bearing wild-type p53 (wt-p53). One of the most frequent inactivation mechanisms of wt-p53 is the overexpression of its negative regulators, MDM2 and MDM4 (also MDMX). These two proteins form a heterodimer that controls p53 activity and levels. In addition, the two proteins function singularly towards p53 with different outcomes depending on the tissue (14, 15). Given its relevance, re-activation of wt-p53 oncosuppressive activity is a field of intense study. In tumors retaining wt-p53, most of the approaches target the inhibitory proteins MDM2/MDM4, either singularly or in combination [reviewed in (16, 17)]. These approaches apply mainly to solid tumors as myeloma, melanoma, and liposarcoma, although some trials have also been applied to acute myeloid leukemia (AML) (for details, see *Therapies Directed to wt-p53 Re-Activation*). Unfortunately, at present, none of these therapeutic approaches have reached the patient’s bed.

Over time, different studies have demonstrated crosstalk between p53/MDM2/MDM4 and sex. Although p53 and its regulators MDM2 and MDM4 genes reside on autosomes, genes associated with p53 circuitry reside on the X chromosome, affecting its function in a sex-related manner. Moreover, genetic variations in the p53/MDM2/MDM4 genes (in the promoter, 3′UTR region, introns, or coding sequence) are responsive to hormone status, affecting p53/MDM4/MDM2 levels and function. Despite all these data, sex differences are not consistently evaluated in preclinical and clinical trials targeting the p53 circuitry.

In this review, we summarize data regarding sex-related factors associated with wt-p53, MDM2, and MDM4. Particularly, we recapitulate the sex-related factors that can affect p53 function by acting on p53 itself or its regulators MDM2 and MDM4. Moreover, we will review sex differences in therapeutic approaches aimed to reactivate wt-p53 and discuss how the consideration of sex could affect the results of these approaches.

## Effects of Sex on p53 Circuitry

### p53

Given the importance of the oncosuppressor p53 in counteracting cancer development, many studies investigated the association of p53 status with sex-related genetic factors and the potential effect of sex-related factors on p53 function.

#### Genetic Factors

A direct association of p53 status with sex-associated cancer difference has been reported in various tumors. In lung cancer, p53 mutations are more frequent in females than in males [reviewed in (8)]. Also, females carrying mutant p53 have an increased risk of developing adrenocortical carcinoma (ACC), suggesting that the p53 oncosuppressive activity impacts ACC development more strongly in females than in males (18). Since ACC risk is also increased in females of pediatric age (19), factors other than sex hormones likely affect p53 function in ACC in a sex-related way (5). At present, these factors have not been identified.

In exon 4 of the p53 gene, the SNP-R72P is present (**Table 1**). The R72 variant possesses higher ability to induce apoptosis compared to P72 [reviewed in (20)]. A hospital-based case–control study of hepatocellular carcinoma (HCC) development in a Turkish population demonstrated that the P72 homozygote (p53^Pro/Pro^) is associated with increased HCC risk in males but not in females (21). A similar association of the variant alleles (P/R + P/P) has been found with a particular squamous cell carcinoma (Kangri cancer) in Indian male subjects (22). These studies suggest that the penetrance/efficacy of the p53^Pro/Pro^ variant is different among male and female subjects. Which factors, genetic or hormonal, alter the p53^Pro/Pro^ activity is currently unknown. It has been proposed that biallelic expression of X-linked tumor-suppressor genes in females explains a portion of the reduced cancer incidence in females compared to males across various tumor types (4). Recently, Haupt and collaborators reported X-linked genes associated with the p53 network. Starting from bio-informatic analyses, they showed that in many non-reproductive cancer histotypes, p53 mutation is more frequent in males than in females with a concomitant lower survival rate (23). Then, they identified X-linked genes encoding for proteins essential for genomic fidelity that are connected to p53. Due to the chromosome X inactivation, females are protected from these gene germline mutations, while males are exposed to a higher risk because they have only a single copy of the X chromosome. The underlying hypothesis is that this link brings to enhanced selection of p53 inactivation in men. This phenomenon is not evident in hormone-dependent tumors since in male breast cancer the frequency of p53 inactivation is reduced compared to females (24). Molecular proof of the ability of these X-linked genes to confer p53-mediated increased protection from cancer in the female gender has not yet been provided.

**Table 1:** Summary of p53, MDM2, MDM4 SNPs relevant to cancer in a sex/hormone-related way.

Gene name	SNP	Variant	Location	Genetic consequence	Cytogenetic region	Alleles	Minor allele	MAF (minor allele frequency)^§^	SNP-linked cancer*
P53	SNP-R72P	rs1042522	chr17:7676154	Missense variant	17p13.1	G>A/G> C/G>T	G	G = 0.4571 (1,000G)	Hepatocellular carcinoma (HCC), squamous cell carcinoma (Kangri cancer)
MDM2	SNP309	rs2279744	chr12:68808800	Intron variant	12q15	T>G	G	G = 0.3666 (1,000G)	Diffuse large B cell lymphoma, soft tissue sarcoma, ER+ invasive ductal adenocarcinoma (IDC), colorectal cancer, pancreatic ductal adenocarcinoma (PDAC), skin squamous cell carcinoma
MDM2	SNP285	rs117039649	chr12:68808776	Intron variant	12q15	G>C	C	C = 0.0134 (1 000G)	
MDM2	SNP55	rs2870820	chr12:68808546	Intron variant	12q15	C>T	T	T = 0.2322	Colon cancer
MDM4	SNP34091	rs4245739	chr1:204549714	3’ UTR	1q32.1	C>A/C>G/C>T	C	C = 0.2141 (1,000G)	ER^-^ breast cancer, prostate cancer, ovarian cancers
MDM4	SNPrs2290854	rs2290854	chr1:204546897	Intron variant	1q32.1	A>C/A>G	A	A = 0.4607 (1,000G)	BRCA-1-associated breast cancer
MDM4	SNPrs4252707	rs4252707	chr1:204539019	Intron variant	1q32.1	G>A	A	A = 0.2196	Glioma

^§^MAF (minor allele frequency) from 1,000 Genomes from 26 different populations divided into five super populations: African, Ad Mixed American, East Asian, European, South Asian; *Tumor histotypes in which the SNP is associated with increased cancer risk.

#### Hormone Activity

Many studies investigated the effect of sex hormones, especially estrogen, on p53 function (25). The picture deriving from these studies is very complex, in some cases reporting opposite results. An important factor that may contribute to this inconsistency is the type of estrogen receptor involved. Indeed, estrogen activity is mediated by the membrane-bound G protein-coupled estrogen receptor 1 (GPER or GP3R0) and the nuclear estrogen receptors (ERs) *α* and *β* (ER*α*, ER*β*), with ER*β* often exhibiting opposite activity to ER*α*. Hormone-stimulated nuclear ERs translocate into the nucleus and direct transcription as homo- and heterodimers or as partners of other transcription factors (indirect genomic signaling) (26). Additionally, the two nuclear receptor genes (named ESR1 and ESR2 corresponding to ER*α* and ER*β*, respectively) originate alternative forms that can interact with the full-length receptors and repress their function. Therefore, the studies performed by stimulation with 17*β*-estradiol (E2, the primary estrogenic hormone that interacts with all estrogen receptors) without characterization of the receptor and the ER*α* and ER*β* isoforms present in the system can be variously interpreted. An additional factor that adds complexity to the interpretation of estrogen activity towards p53 is the positive regulation of MDM2 levels by estrogens (see relative paragraph *MDM2*). Since MDM2 is a negative regulator of p53, the fine-tuning of MDM2 and p53 by estrogens can result in different outcomes.

Since ER*α* is a critical therapeutic target in hormone-responsive breast and endometrial cancers, many data described in literature investigated the interplay between p53 and ER*α*. Particularly, since p53 mutation is not common in estrogen-responsive breast cancer, accounting for about 20% of tumors (27, 28), many studies focused on this tissue.

Two main and opposite estrogenic hormone activities towards p53 have been described: a positive activity at different levels and a repressive activity mainly related to p53-transcriptional function.

##### Cooperative Estrogen-p53 Activity

As concerns the mechanism of cooperation, there are various studies from Olivier’s group (29–31). In cell lines derived from breast cancer MCF7 cells, they demonstrated that estrogen through endogenous ER*α* increases p53 levels and enhances p53-mediated response to DNA damage. They further showed that focal adhesion kinase (FAK), a critical regulator of adhesion and motility, is downregulated by p53 in response to E2 (31). These studies have been further confirmed by Berger and colleagues, who demonstrated that the p53 promoter contains four ER*α* responsive elements (ERE) (32). Accordingly, knockdown of ER*α* leads to decreased p53 mRNA and protein levels and its targets, MDM2 and p21. This results in increased colony formation in an estrogen-free medium upon a cytostatic dose (100–400 nM) of doxorubicin (32). Of note, these authors demonstrate that the ER*β* receptor is not involved since its exogenous expression does not alter p53 levels and activity. In support of this data, Klaus and colleagues analyzed the radiation-responsiveness of the mammary epithelium in ovariectomized mice upon E2 and progesterone (P) treatment (33). These hormones activate the p53 response to radiation in terms of increased p21. Also, by comparing the mammary epithelium of BALB/c mice with different p53 status (Trp53^+/+^, Trp53^+/−^, Trp53^−/−^), they demonstrated that E + P upregulated p53 nuclear levels and apoptosis upon ionizing radiation, also in the haploinsufficient background (BALB/c Trp53^+^/^−^) (34). Interestingly, parity acted similarly and delayed the onset of spontaneous mammary tumors in these mice, confirming a protective role of hormones towards breast cancer development. Similar data were obtained by Sivaraman using the rat model (35). Importantly, epidemiologic studies show that women with full-term pregnancy have a significantly reduced risk of developing ER^+^ breast cancer (36). In agreement with these studies, Kupperwasser and colleagues reported that mare serum gonadotropin (PMSG) and human gonadotropin (hCG) treatment increases p53 nuclear fraction leading to enhanced mammary gland apoptosis following ionizing radiation (37). Interestingly, a recent proteome analysis of MCF-7 cells demonstrated that estrogen modulates cyto-nuclear shuttling; in response to estrogen, dynamic subcellular redistribution of proteins is the major phenomenon compared to the alteration of protein levels (38). Overall, this data strongly supports that estrogen enhances the oncosuppressive function of p53 in the breast tissue. Also, in another normal epithelial context as Young Adult Mouse Colon cells (YAMC), estrogen induces p53 downstream targets as PUMA, Bcl-2-associated X protein (Bax), and Noxa, and sensitizes cells to p53-mediated apoptosis (39), extending the cooperative function of p53 and estrogen in another epithelium. The good prognosis of ER*α* ^+^/wt-p53 breast cancer can also be related to a cooperative activity between these two factors. To integrate this hypothesis, p53 inhibits ER*α* transcriptional activity on synthetic estrogen-responsive elements (40), suggesting a tumor-suppressive function of p53 towards ER*α* in hormone-activated signaling pathways.

This data raises a question about the consequences on p53 of anti-estrogenic therapies in breast cancer (41). Since these drugs antagonize ER function, they should reduce p53 oncosuppressive activity. The observation that anti-estrogenic therapy displays partial agonist activity in a gene-specific and tissue-specific manner partly solves this issue (42). In this regard, Olivier’s group demonstrated that 4-hydroxy-tamoxifen (OHT), a selective estrogen receptor modulator (SERM), suppresses cell proliferation more effectively in breast cancer cell lines bearing wild-type p53 compared to cells with mutated p53. Furthermore, p53 expression levels have been reported as a positive prognostic factor for OHT treatment (43). Conversely, the activity of fulvestrant (ICI 182,780), a selective estrogen receptor degrader (SERD) that acts by inducing degradation of nuclear estrogen receptors is independent of p53 status (30). This data suggests that the p53-estrogen crosstalk is differently affected by estrogen, OHT, and fulvestrant, and supports the efficacy of anti-estrogenic therapies in wt-p53 breast cancer.

By considering hepatic tissue, Pok and colleagues showed that testosterone positively regulates hepatocyte cell cycle regulators and reduces p53 and p21, while E2 plays the opposite effect (44). Accordingly, in liver cancer cell lines, E2, *via* ER*α*, activates the transcription of p53 and its target miR-23a, promoting p53-dependent apoptosis and, in turn, inhibiting HCC development (45). Since men are more susceptible than women to hepatocellular carcinoma (HCC) at age <60 years (46), this data supports a protective role of estrogenic hormones in liver cancer risk and highlights a possible mechanism by which sex hormones contribute to establishing the male prevalence of hepatocarcinoma.

Few studies analyzed the effects of ER*β* on p53. In colon cancer cell lines, ER*β* overexpression enhances p53 levels and activity through p14ARF-mediated downregulation of MDM2. Of note, this cell outcome was observed in many but not all colon cancer cell lines analyzed. The reason for these results remains unexplained (47). Similarly, in the human colon metastatic LoVo cell line, overexpression of ER*β* enhances p53-mediated apoptosis in an estrogen-dependent manner (48). Overall, these studies indicate a proapoptotic function and anti-oncogenic activity of ER*β* towards p53 although in tissues other than the breast.

##### Antagonistic Estrogen-p53 Activity

Opposite to this view, Das’s group reported that ER*α* inhibits p53 function on some transcriptional targets (49). The model proposed by these authors is that ER*α* and p53 cooperatively bind on the promoters of some p53-targets genes at whose levels ER*α* represses p53 activity. In most of these studies, cell outcome is not reported, so they are not entirely comparable to previous studies. One limitation of these studies is that they are often based on ER*α* overexpression. Indeed, in MCF7 without ER*α* overexpression, endogenous ER*α* does not affect p53 transcriptional function following E2 or fulvestrant treatments (50). A further explanation can derive from studies of Brown’s group (51). They demonstrated in MCF7 that E2 and OHT reduce the apoptosis induced by cytotoxic high dose of doxorubicin (10 μM) whereas fulvestrant is inefficacious. Using genome-wide approaches, they reported the modulation of a subset of p53 and ER target genes, but not changes of p53 levels and its binding to these gene promoters. This data suggests that different p53 targets can be variously regulated upon specific estrogen treatments, leading to different cell outcomes. Also, Lewandowski and collaborators reported an antagonistic activity of estrogen towards p53. In MCF7 cells, E2, through ER*α*, mediates the relocalization of p53 from the nucleus to the cytoplasm, inhibiting its transcriptional activity as revealed by decreased p21 levels. This, in turn, results in reduced sensitivity of MCF7 to TNF-mediated cell death while ER*β* behaves oppositely, antagonizing cytoplasmic relocalization of p53 (52). Interestingly, a study from Bargonetti’s group demonstrated that in MCF7, estrogen-induced cell proliferation and downregulation of p21 are p53-independent but MDM2 dependent (53). Therefore, E2-induced p21 modulation as a marker of p53 activity can be misleading. Moreover, the different crosstalk between ER*α* and p53 could also be ascribed to specific treatments, such as cytostatic (32) *vs.* cytotoxic (49) doses of doxorubicin, *γ*-irradiation (33), or TNFα (51).

### MDM2

MDM2 protein is involved in a negative feedback loop with p53, by which p53 activates transcription of the MDM2 oncogene, which in turn inhibits p53 activity. This loop is essential to maintain both proteins at moderate levels and reset cell behavior after p53 activation. Due to their intertwined role, an unbalanced expression or activity of MDM2 is involved in cancer, and many studies highlighted sex-related factors leading to unbalanced MDM2 (15). Additionally, MDM2 regulates targets other than p53, which also have relevance to cancer (54).

#### Genetic Factors

The expression of the MDM2 gene is probably the best example of sex-mediated regulation of p53 circuitry in cancer. An initial report from Blaydes’s group demonstrated the activation of the MDM2 P2 promoter by the AP1-ETS transcription factors in an ER*α* dependent manner (55). Subsequently, Bond and colleagues identified the SNP309 T/G within this promoter (56) (**Table 1**). The SNP309G variant extends the length of the DNA binding site for specificity protein 1 (Sp1), increasing the affinity for this transcriptional factor. As a result, Sp1 increases MDM2 levels, leading to an attenuation of the oncosuppressive p53 activity. This SNP is indeed associated with an early age of cancer diagnosis. Since Sp1 is a co-transcriptional factor of ERs, the authors demonstrated that SNP309 accelerates the age of onset of various cancer types (diffuse large B cell lymphoma, soft tissue sarcoma, invasive ductal breast carcinoma, IDC)—in female but not in male patients (57). Accordingly, this sex difference is associated with ER^+^ but not ER^−^ invasive ductal breast carcinoma and is more evident in non-menopausal women. Similar data were reported for colorectal cancer, in which female SNP309G carriers were diagnosed with cancer earlier than those carrying the wild-type gene (58). Other studies evidenced the relevance of this SNP in a p53-independent way due to the ubiquitin ligase activity of MDM2 towards other targets (53, 59, 60). Overall, this data underlies the role of the estrogen-mediated pathway on MDM2 function through SNP309. At odds with this data, some studies did not find an association between SNP309 and estrogen status on cancer risk [reviewed in (61)]. Although, in many cases, the authors did not take into account the sex and the hormone levels, a resolving study from Lønning’s group defined the presence of the additional SNP285G>C, which antagonizes the Sp1 binding to SNP309 (**Table 1**) (62). The presence of this SNP reduces the risk of both ovarian and breast cancers, highlighting the relevance of MDM2 fine-tuning for cancer development.

Further complexity has been recently added by Lozano’s group, who demonstrated that MDM2 SNP309G exhibits tissue-specific regulation and different impacts on cancer risk (63). Accordingly, Grochola and colleagues showed that in pancreatic ductal adenocarcinoma (PDAC), the SNP309G is associated with earlier onset in men but not in women. They attributed this effect to the function of Sp1 as a coactivator of androgen receptors present in PDAC (64).

In 2015, Kato’s group identified an additional SNP in MDM2-P2 promoter, the SNP55 (rs2870820, C/T) (65). Both SNP55T and SNP55C bind Sp1, whereas only the C allelic variant creates an additional consensus sequence for the transcriptional factor NF-kB. The NF-kB p50/p50 homodimer interferes with Sp1 transcriptional activity, as demonstrated by Hirano and colleagues (66). In the context of MDM2, this results in transcriptional repression of the gene (65). Therefore, this SNP further contributes to fine-tuning MDM2 levels. Subsequently, Helwa and colleagues reported that women with SNP55TT or SNP55TC genotype have a higher risk of colon cancer, particularly left-sided colon cancer, than those with SNP55CC genotype (66). Conversely, this SNP does not seem to affect breast, lung, prostate, and endometrial cancer risk (66). In a recent study, the same group analyzed the impact of the combination of all three SNPs and reported that the SNP55T allele variant is associated with a lower risk of endometrial cancer in women carrying the SNP285G and SNP309T. At the same time, this haplotype is not correlated with the risk of ovarian cancer (67).

These results collectively validate the MDM2 SNPs as important cancer modifiers by attenuating the cell-protective activity of p53 or p53-independent pathways. To date, the characterization of these SNPs in the application of MDM2-target therapies has not been reported.

#### Hormone Activity

Besides the effect of the hormone on MDM2 transcription through SNPs, other studies evidenced a regulation of MDM2 by the estrogenic pathway at the protein levels. ER*α* stabilizes MDM2 since the use of fulvestrant significantly reduces the MDM2 half-life. Particularly, fulvestrant decreased basal expression of MDM2 through increased protein turnover in the absence of E2 (68). This in turn, increases cell apoptosis and sensitivity of MCF7 breast cancer cells to chemotherapic drugs, doxorubicin, paclitaxel, and etoposide (68). Accordingly, high levels of MDM2 are detected in ER*α* ^+^ breast carcinoma (69). In a reciprocal fashion, Cavailles’s group demonstrated MDM2 activity towards ER*α*: MDM2 interacts with ER*α* and p53 and induces ER*α* degradation through its ubiquitin ligase activity in a ligand-independent manner (70). Conversely, in a p53-independent way, Bargonetti’s group reported the ability of MDM2 to facilitate the estrogen-mediated activation of cell proliferation (53), suggesting different activities of MDM2 dependent on p53 background.

The overall positive effects of estrogenic hormones on MDM2, at the mRNA and protein levels, suggest that anti-estrogenic therapies in breast cancer could synergize with MDM2-targeted drugs.

### MDM4

MDM4 is a double-faced p53 regulator: it cooperates with MDM2 in inhibiting p53, thus behaving as an oncogenic factor. Conversely, under DNA damage conditions, it cooperates with p53 and promotes cell apoptosis (71, 72). MDM4 also possesses a p53-independent function by suppressing the mTOR-mediated pathway (73, 74). Of relevance, the proapoptotic activities reside on the cytoplasmic fraction of MDM4 (75, 76). Accordingly, most tumors show high levels of MDM4 in the nuclear compartment (77). Wide-genome studies reported the association of specific SNPs in the MDM4 gene with hormone-mediated cancer and estrogen receptor-negative breast tumors, suggesting that the presence of ERs may select for a particular MDM4 gene status.

#### Genetic Factors

Despite the description of various SNPs in the MDM4 gene (78), the majority of data focused on the SNP 34091 (A > C) in the 3′UTR region of human MDM4 (**Table 1**). Data collected from the Collaborative Oncological Gene-environment (COGS) showed a significant association of this SNP with hormone-dependent cancers (79–81). This SNP located 32 bp downstream of the stop codon should create an illegitimate binding site for miR-887 (80, 82) and has-miR-191, a miRNA often expressed in tumor tissues, leading to downregulation of MDM4 in the MDM4-C variant (83). Unexpectedly, the SNP34091C variant is associated with increased risk of breast cancer, high-grade ovarian cancer (HGSOC), and prostate cancer suggesting a specific sensitivity of these hormone-dependent cancers to presumably low MDM4 levels (79, 84). SNP34091C is associated with increased cancer risk only in ER-negative breast cancer, suggesting that the presence of ERs interferes with MDM4 activity (79, 85). Additionally, in ER-negative breast cancer and HGSOC, the presence of this SNP is not correlated to the status of p53, suggesting that ERs interfere with MDM4 p53-independent activities. This data is in agreement with the cytoplasmic proapoptotic function of MDM4. The ER ability to re-localize MDM4 in the nucleus would abrogate the MDM4 cytoplasmic anti-oncogenic function. Accordingly, many human tumors express nuclear MDM4 (77). Other studies on ovarian cancer reported contradictory results. Wynendaele reported an association of the AA haplotype with reduced overall survival of ovarian carcinoma. Conversely, Gansmo reported an association of C variant with HGSOC (84). A possible explanation raised by these last authors is that in HGSOC, p53 is mutated in almost 90% of tumors. Therefore, the effect of this variant is mediated *via* pathways other than p53 (84).

The association between hormone-related pathways and this SNP is supported by the observation that it does not alter colon- and lung-cancer risk in a large population-based control study (86). Of merit, this study distinguished male and female patients compared to male and female controls. Association with prostate cancer was not found, although some authors reported a trend of association of the C allele with higher prostate cancer aggressiveness (87).

At odds with the previous study, other authors reported a reduced cancer risk for the SNP34091C variant in esophageal squamous cell carcinoma and non-Hodgkin lymphoma (88, 89). Information on ERs status in these studies is not available and cannot be entirely compared to previous results. Additionally, the different geographic populations (Caucasian *vs.* Chinese) may underlie this discrepancy. Indeed, the frequency of the MDM4-SNP34091 is different between these two populations (90). Finally, other miRNAs may affect SNP function (91).

Additional SNPs have been identified in other cancer types. Couch and colleagues reported the association of the minor allele of MDM4 SNP rs2290854 with breast cancer risk in mutant BRCA1 carriers, suggesting that this MDM4 variant can be a modifying factor for breast cancer in this mutant background (92) (**Table 1**). Also, in this case, this SNP is associated with ER^−^ but not ER^+^ breast cancer. Moreover, two recent papers analyzed the sex-related association of various MDM4 SNP with glioma in the European and Chinese populations (93, 94). The authors evidenced the association of the A allele of a novel MDM4 SNP (rs4252707) (**Table 1**) with the increased risk of this tumor. However, although the higher frequency of glioma in males, they did not find an association with sex.

#### Hormone Activity

Lozano’s group was the first to demonstrate a dissimilar activity of overexpressed MDM4 between male and female mice (95). Her group reported a higher incidence of multiple tumors and a decreased animal survival in Mdm4 transgenic p53-null males but not in females, indicating sexual dimorphism of Mdm4 activity, at least in rodents. Using the same animal model, our group demonstrated that in a wt-p53 background, Mdm4 promotes tumor development following DNA damage in a sex-independent way, indicating that Mdm4 oncogenic properties are not affected by sex. In contrast, there is increased chemotherapy sensitivity in Mdm4-overexpressing male but not in female mice. Molecular analysis demonstrated that E2 re-localizes MDM4 in the nucleus, antagonizing the cytoplasmic MDM4-mediated DNA damage response (96). Noteworthy, treatment of animals with fulvestrant rescues MDM4-mediated proapoptotic activity and increases tumor sensitivity to chemotherapy. Accordingly, MDM4 nuclear localization and intra-tumor estrogen availability correlate with decreased platinum sensitivity and apoptosis and predict poor disease-free survival in human HGSOC. A specular finding was reported by Das’s group (97). They showed that ER*α* is a positive regulator of MDM4 oncogenic activity, and treatment of tumor cells with ER inhibitors (fulvestrant or OHT) reduces MDM4 protein levels. Overall, this data indicates that sex and/or ER*α* regulate MDM4 activity. Depending on p53 background, they can result in opposite outcomes. The assessment of sex/hormone status in MDM4-target therapy could confirm the relevance of these factors in drug efficacy and suggest the potential usefulness of combinatorial treatments.

## Therapies Directed to wt-p53 Re-Activation

In the last decades, different therapeutic approaches were developed to reactivate wt-p53 functions in cancer. Peptides and small molecule compounds were described to target the critical inhibitory binding of MDM2 and MDM4 to p53 or to stimulate p53 by acting on the protein itself [reviewed in (16, 98, 99)].

Compounds in the p53 network that entered clinical trials are summarized in **Table 2**. Among them, the majority refers to phase I studies that evaluate safety and tolerability, with few or no results about the efficacy of the therapies. Despite the low number of patients usually enrolled in phase I, none of these trials reported the hormone/gender status in their evaluations. Here, we briefly reviewed the active clinical trials suggesting the potential role of sex/hormone.

**Table 2:** Strategies for wt-p53 re-activation, which entered clinical trial phases.

Compound	Drug	Mechanism of action	Clinical trial ID (Phase)
**p28**	Peptide	Inhibition of COP1-mediated p53 ubiquitination	NCT00914914 (I), NCT01975116 (I)
**ALRN-6924**	Peptide	Dual inhibition of MDM2 and MDM4 interaction with p53	NCT02264613 (I,IIa), NCT03725436 (I), NCT02909972 (I,Ib), NCT03654716 (I)
**RG7112 RO5045337**	Small molecule	Binds MDM2 and prevents MDM2/P53 interaction	NCT01677780 (I), NCT01164033(I), NCT01143740(I), NCT01605526(I), NCT00623870(I), NCT00559533(I), NCT01635296(I)
**RG7388 RO5503781**	Small molecule	Binds MDM2 and prevents MDM2/P53 interaction	NCT02407080 (I), NCT02828930 (I), NCT02633059 (I,II), NCT02545283 (III), NCT02624986 (Ib, II), NCT01901172 (I), NCT01773408 (I), NCT01462175 (I)
**SAR405838 MI-77301**	Small molecule	Mimics p53 key amino acids involved in MDM2 binding	NCT01636479 (I), NCT01985191 (I)
**CGM097**	Small molecule	Mimics p53 key amino acids involved in MDM2 binding	NCT01760525 (I)
**MK-8242 SCH 900242**	Small molecule	Inhibition of MDM2 interaction with p53	NCT01463696 (I), NCT01451437 (I)
**AMG232**	Small molecule	Inhibition of MDM2 interaction with p53	NCT02016729 (Ib), NCT03031730 (I), NCT01723020 (I), NCT02110355 (Ib,IIa)
**DS-3032b**	Small molecule	Inhibition of MDM2 interaction with p53	NCT02579824 (I), NCT02319369 (I), NCT01877382 (I)
**HDM201**	Small molecule	Inhibition of MDM2 interaction with p53	NCT02343172 (Ib,II), NCT02143635 (I), NCT02780128 (I), NCT02601378 (I), NCT03940352 (I), NCT03714958 (I), NCT04496999 (I), NCT04116541 (II), NCT04097821 (I,II), NCT02890069 (I)
**Data from ClinicalTrials.gov. National Library of Medicine:** http://www.clinicaltrials.gov			

P28 is a peptide derived from the Azurin, a *Pseudomonas aeruginosa* redox protein that exerts an antiproliferative activity towards cancer cells by inhibiting COP-1 mediated ubiquitination of p53, thus in an MDM2/MDM4 independent way (100, 101). In a Phase I study (NCT00914914), p28 proved preliminary evidence of anti-tumor activity in patients with melanoma and colon cancer (**Table 2**). Although no results regarding gender are displayed (102), P28 efficacy towards these non-hormone mediated cancers might suggest that COP-1 mediated regulation of p53 is not affected by hormone status. Accordingly, a second Phase I study NCT01975116 established safety in children with recurrent or refractory central nervous system cancer (CNS) (103). Comparing this peptide efficacy in males and females could highlight the potential effects of genetic factor/s on p53 function.

ALRN-6924 is a peptide that targets both MDM2 and MDM4 and prevents their binding to p53 (104). Phase I studies evaluated safety in acute myeloid leukemia (AML), myelodysplastic syndrome (MDS), and solid cancers (**Table 2**). Two recent trials are recruiting patients to evaluate the safety and efficacy of ALRN-6924 in combination with drugs used in chemotherapy as Cytarabine for patients with leukemia (NCT03654716) or Paclitaxel for those with breast cancer (NCT03725436). The results of this last trial could be of interest to evaluate the relevance of ERs in p53 circuitry since the inclusion criteria are breast cancer carrying wt-p53 and positive for ERs.

RG7112 and RG7388, two derivatives of cis-imidazoline molecule known as “nutlin”, are under testing in clinical trials (**Table 2**). RG7112, despite promising results in terms of p53 activation, was dropped because of significant toxicity (105). RG7388, known as Idasanutlin, is a nutlin analog with a higher affinity and specificity for MDM2. This compound underwent several clinical trials, including phase II and phase III trials in combination with chemotherapy or novel therapies as monoclonal antibodies and other new anticancer small molecules (**Table 2**). Based on the relevance of ER*α* in MDM2 levels, the efficacy of this drug could be increased in those tumors previously shown more sensitive to SNP variations.

Spirooxindole-based compound mimics the p53 key amino acids that bind MDM2. MI-77301 from Sanofi (106) and the substituted dihydroisoquinolinone derivative CGM097 from Novartis, entered in phase I clinical trials (**Table 2**). For both compounds, the anti-tumor activity has been verified in preclinical studies (107), whereas no results have been reported from clinical trials. Of interest, p53 status is not sufficient to predict CGM097 sensitivity in a panel of 477 cell lines from the Cancer Cell Line Encyclopedia (CCLE). In the 13 gene signature predicting CGM097 response, MDM2 levels are the most significant predictor (108). Given the relevance of ER/hormone status in affecting MDM2 levels, the assessment of hormone status could be relevant in the analysis of the efficacy of these compounds in the related clinical trials.

MK-8242 from Merck is a small-molecules that inhibits MDM2 interaction with p53 and can induce growth arrest at very low concentration (109). In the NCT01463696 trial, three of 47 patients with liposarcoma showed a partial response, and 31 patients stable disease (110) (**Table 2**). Since 60% of these patients are males, it would be interesting to re-evaluate the results by separate analysis of patients based on sex/hormone status.

AMG232 by Amgen is a piperidinone-derived compound that acts as a potent inhibitor of the MDM2−p53 complex and shows high anti-tumor activity in xenograft models (111). This compound underwent several clinical trials, including phases I and II, as single or combination treatments for solid tumors, AML, myeloma, and melanoma (**Table 2**), but its effects have never been evaluated in the light of sex/hormones.

DS3032b developed by Daiichi Sankyo showed stable disease in 77% of patients with solid tumors (112) and reduced bone marrow blasts after the first cycle in half patients and complete remission in two patients with hematological malignancies.

HDM201 developed by Novartis is an imidazopyrrolidinone analog that inhibits the P53–MDM2 interaction with high efficiency. It was able to induce p53 dependent apoptosis and tumor regression in xenograft tumor models (113). Many clinical studies are ongoing in patients with wild-type p53 tumors of different histotypes such as AML, solid tumors, and multiple myeloma (**Table 2**). Some results of clinical benefit from these trials have been reported: approximately 25–30% of patients had a partial response or stable disease, although the tumor histotypes are not specified (114).

Many of the tumors under these clinical trials—including myeloma and lymphoma—show sex differences, with male prevalence. Evaluating these drugs in terms of sex and/or hormone status could give valuable information for more personalized medicine.

## Discussion

Although sex is an important factor in determining cancer development, progression, and sensitivity to therapy, sex-based studies of cancer biology and treatment are still largely insufficient, and the factors driving the sex-related cancer disparity remain to be clarified. Even after recommendations from NIH and other funding agencies to consider sex and gender at all levels of biomedical research, animal studies and clinical trials that distinguish gender populations are few (115). P53-target therapies do not make an exception, as demonstrated by **Table 2**. None of those clinical trials reported separate results for men and women or considered the hormonal status of patients. Still, many studies demonstrate that p53 activity is affected by sex-related genetic and/or hormone determinants. This review reflects the abundance and complexity of the sex-related molecular factors that affect p53 response in human tumors. Based on the data here reviewed, nowadays, it is difficult to predict which genetic or hormonal factors could contribute to defining a more personalized application of single or combinatorial treatments.

For this reason, evaluation of sex/hormones in preclinical studies and clinical trials could help clarify the factors that finally affect p53 function *in vivo* and could guide future molecular studies besides drive a more appropriate and successful application of these therapies. Indeed, “false” negative or positive results could be due to the confounding effects of mixed backgrounds. As stated by Clayton and Collins, “inadequate inclusion of female cells and animals in experiments and inadequate analysis of data by sex may well contribute to the troubling rise of reproducibility in preclinical biomedical research” (115).

Inclusion of sex/hormone status in the analysis of p53 data could open the possibility of combinatorial treatments with anti-hormone or other target therapies. Based on the beneficial effect of SERM on tumors with wt-p53 and possible depressing activity of MDM2 levels, a combinatorial treatment of SERM therapy with p53-reactivating drugs in breast cancer could be hypothesized. Accordingly, Rozeboom and colleagues recently suggested a new clinical trial based on triple therapy with a BCL2 inhibitor (venetoclax), an anti-estrogen (tamoxifen/fulvestrant), and an MDM2 inhibitor (AMG-232/MI-77301) in the ER^+^/WT *TP53* metastatic breast cancer setting (116). Finally, the ascertainment of sex/hormone and p53 crosstalk could guide different drug dosages and improve safety-toxicity drug features. This is particularly relevant given the more active immune response in females than males and the well-known required lower doses of heart disease drugs in females compared to men (117, 118).

## Author Contributions

FMa, LG, ET, and FMo wrote the manuscript. AP revised the manuscript. FMo conceived and reviewed the manuscript. All authors contributed to the article and approved the submitted version.

## Funding

This study was supported by grant from Italian Association for Cancer Research (AIRC) under IG 2018—ID. 21814 project—P.I. Moretti Fabiola.

## Conflict of Interest

The authors declare that the research was conducted in the absence of any commercial or financial relationships that could be construed as a potential conflict of interest.
